# Supplementary material for: Rumenomics: Evaluation of rumen metabolites from healthy sheep identifies differentially produced metabolites across sex, age, and weight
Source: bioRxiv. 2025 Feb 8:2025.02.05.636747. Preprint. [Version 1] doi: 10.1101/2025.02.05.636747 (PMC11839056; doi:10.1101/2025.02.05.636747)
Supplement: Supplement 2 [file NIHPP2025.02.05.636747v1-supplement-2.pdf]

bioRxiv preprint doi: https://doi.org/10.1101/2025.02.05.636747; this version posted February 8, 2025. The copyright holder for this preprint (which was not certified by peer review) is the author/funder, who has granted bioRxiv a license to display the preprint in perpetuity. It is made available under aCC-BY 4.0 International license.

SUPPLEMENTAL FIGURES

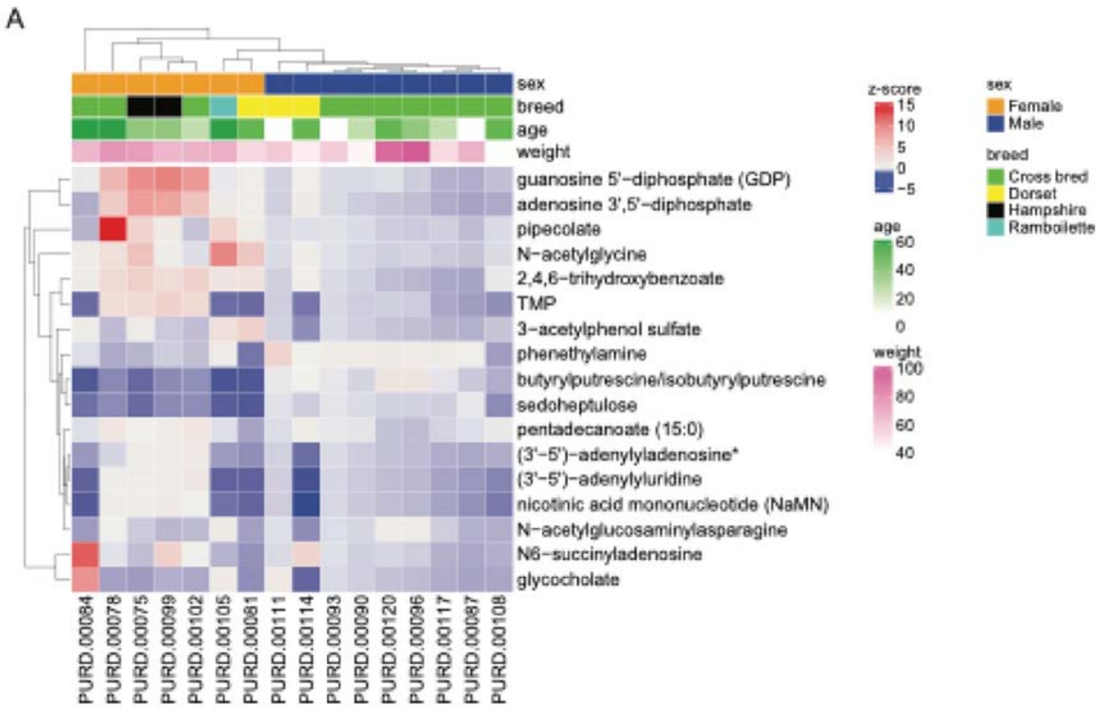

Figure S1. Heatmap of significant metabolites (p<0.05) based on univariate linear modeling identified metabolites associated with sheep sex, weight, and age.

bioRxiv preprint doi: https://doi.org/10.1101/2025.02.05.636747; this version posted February 8, 2025. The copyright holder for this preprint (which was not certified by peer review) is the author/funder, who has granted bioRxiv a license to display the preprint in perpetuity. It is made available under aCC-BY 4.0 International license.

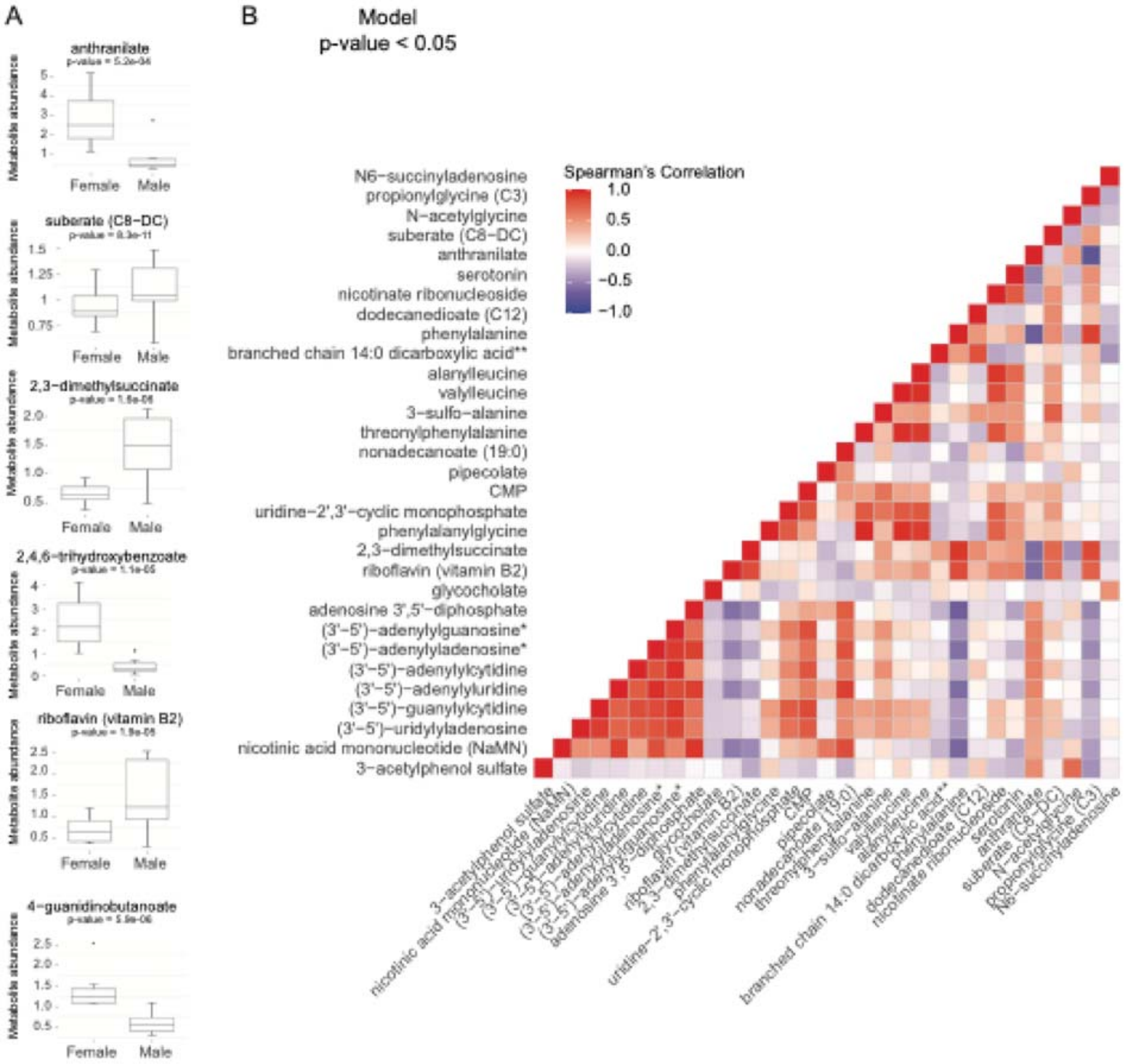

Figure S2. Prioritized metabolites from interaction effects univariate linear modeling. (A) Boxplots of sheep sex-based differences of anthranilate, suberate (C8-DC), 2,3-dimethylsuccinate, 2,4,6-trihydroxybenzoate, riboflavin (vitamin B12), and 4-guanidinobutanoate (p-value < 0.05). (B) Spearman's correlation analysis from metabolites with model p-values < 0.05.
